# Quantitating Fluorescence Intensity From Fluorophores: Practical Use of MESF Values

**DOI:** 10.6028/jres.107.027

**Published:** 2002-08-01

**Authors:** Lili Wang, Adolfas K. Gaigalas, Fatima Abbasi, Gerald E. Marti, Robert F. Vogt, Abe Schwartz

**Affiliations:** National Institute of Standards and Technology, Gaithersburg, MD 20899-8312; Center for Biologics Evaluation and Research, U.S. Food and Drug Administration, NIH Building 29B, Room 2NN08, Bethesda, MD 20892; Division of Laboratory Sciences, CDC, Mailstop F19, 4770 Buford Highway, Atlanta, GA 30341; Center for Quantitative Cytometry, PO Box 194344, San Juan, Puerto Rico 00919

**Keywords:** emission spectrum matching, extinction coefficient, fluorescein, lymphocyte, MESF value, microbead, pH, quantitative flow cytometry

## Abstract

The present work uses fluorescein as the model fluorophore and points out critical steps in the use of MESF (Molecules of Equivalent Soluble Fluorophores) values for quantitative flow cytometric measurements. It has been found that emission spectrum matching between a reference solution and an analyte and normalization by the corresponding extinction coefficient are required for quantifying fluorescence signals using flow cytometers. Because of the use of fluorescein, the pH value of the medium is also critical for accurate MESF assignments. Given that the emission spectrum shapes of microbead suspensions and stained biological cells are not significantly different, the percentage of error due to spectrum mismatch is estimated. We have also found that the emission spectrum of a microbead with a seven-methylene linker between the fluorescein and the bead surface (bead7) provides the best match with the spectra from biological cells. Therefore, bead7 is potentially a better calibration standard for flow cytometers than the existing one that is commercially available and used in the present study.

## 1. Introduction

Quantitative flow cytometry, QFCM, has been advocated for the interlaboratory data comparison and the quality control of clinical flow cytometry for about a decade [1, 2]. It has made it possible to express not only the percentage of positive cells in a sample but also the absolute number of antibodies bound to a single cell. There are, for example, reports demonstrating the feasibility of assessing CD38 expression levels on CD8^+^ T lymphocytes through quantitative cytometric analyses [3–5]. Further applications of quantitative cytometry will depend on some means of standardizing immunophenotyping across a wide range of products in a variety of clinical settings with a variety of possible fluorescence reference material choices.

The present work is a follow-up investigation on the practical use of Molecules of Equivalent Soluble Fluorophore values, MESF, defined and published earlier [6]. The MESF approach relies on the equivalency between the number of fluorophores in two solutions, where one solution may be a suspension of labeled microbeads. To simplify the assignment of MESF values to labeled microbeads in a suspension using a fluorophore solution, for instance, the use of the same fluorophore was suggested. This prerequisite ensures a minimum change in molar absorptivity in both environments. For the same instrument setting, the equality of fluorescence intensities of two solutions or suspensions integrated over entire emission spectra is equivalent to the equality of fluorescence yields of those two solutions or suspensions. The fluorescence yield is a product of the number of fluorophores in solution or the number of labeled beads in suspension and the corresponding fluorescence quantum yield. The MESF value of the bead suspension is the ratio between the number of fluorophores in solution and the number of beads in suspension. The assignments of MESF values to a set of labeled beads with different fluorophore densities allow for construction of a calibration curve for an instrument, such as a flow cytometer. The procedure allows one to obtain MESF values of biological cells stained with the same fluorophore-labeled monoclonal antibodies, the goal of the present study. Practically, if one uses antibodies with known MESF values, which are assigned using the same methodology for obtaining MESF values of beads, the number of antibody binding sites per cell is equal to the ratio of the MESF value per cell and the MESF value per antibody. This number, defined as Antibody Binding Capacity (ABC), is the ultimate biological objective associated with quantitative cytometric measurements.

Although both concepts, MESF and ABC, are well accepted in the flow cytometry community [7], there seems to be a lack of solid foundations as to the limits for the use of MESF values and choice of cytometric reference standards. In the present study, we use Fluorescein Solution SRM^®^ 1932, a NIST fluorescence standard reference solution, as the primary standard, and focus on the spectral properties of beads labeled with fluorescein with different linker lengths, fluorescein-labeled antibodies, and biological cells such as leukocytes and lymphocytes stained with fluorescein-labeled antibodies. We have also used the existing microbead calibration standard to construct calibration curves for MESF assignments of the stained lymphocytes. The assignments were made independently using two different flow cytometers. Given that fluorescein is the only fluorophore used throughout the study, cytometric variations in the MESF values obtained are largely due to both procedure error and systematic error. The former is highly related to the measurement procedures. The later is caused by the spectral mismatch between the bead standard and the stained lymphocytes because of the use of the bandpass filter in the cytometer and wavelength-dependent instrument detection responses as explained below. Mathematical modeling is used to simulate the actual data and to establish the percentage of the systematic error. We demonstrate that such error can be avoided by using a better microbead reference material. This investigation is important for the evaluation of the existing fluorescein-based bead standard and for the establishment of the correct use of MESF values.

## 2. Materials and Methods

### 2.1 Experimental Details

Beads labeled with fluorescein having different linker lengths were obtained from Flow Cytometry Standards Corporation^1^ (San Juan, PR). Quantum™ FITC MESF Kit (medium level), which is composed of a set of microbeads with different amounts of fluorescein labeled via a three-methylene linker to the bead surface, was obtained from Bangs Laboratories, Inc. (Fishers, IN). Monoclonal antibodies CD45, CD3, CD8, and CD45RA, all labeled with fluorescein, were purchased from BD Immunocytometry Systems (San Jose, CA). In addition, 25 different fluorescein-labeled monoclonal antibodies used in this study were provided by Dr. Thomas Fleisher at NIH.

Cells were stained with various monoclonal antibodies followed widely used procedures. Briefly, the whole blood was first washed with 10 % Fetal Bovine Serum in 1X Phosphate Buffered Saline (PBS, pH 7.2, containing 9.0 g/L of NaCl, 0.726 g/L of Na_2_HPO_4_·7H_2_O, and 0.21 g/L of KH_2_PO_4_), and then stained with various fluorescein-labeled antibodies for 30 min at 4 °C. The stained cells were subsequently lysed with 1X FACS™ Lysing Solution and the obtained leukocytes were resuspended in 1 mL of 1 % fixative (Formaldehyde, Electron Microscopy Sciences, Fort Washington, PA) after washing twice with 1X PBS/0.1 % sodium azide.

The mononuclear cells from the whole blood were obtained using the Ficoll-Hypaque separation procedure [8, 9]. A 15 mL quantity of Lymphocyte Separation Medium (ICN Biomedical Inc., Aurora, OH) was gently laid beneath 30 mL of diluted blood in a 50 mL conical centrifuge tube. The tube was spun at 900 g for 5 min. The top plasma layer was aspirated to within 1 mL to 2 mL of the mononuclear cell layer, and the white cell layer was subsequently transferred into a clear tube. A small amount of red cells in the white cell layer was cleaned out by lysing with ammonium chloride followed by centrifuging at 400 g for 10 min. The mononuclear cells were stained with antibodies following the above procedure except the lysing step.

The emission spectra were measured using a custom-made calibrated spectrofluorimeter described previously [6]. The flow cell configuration was adapted such that approximately 6 mL of sample passed through the excitation beam at the rate of 9 mL/min by means of a peristaltic pump from Gilson, Inc., Middleton, WI. The flow system minimizes photodegradation of the fluorophores.

**Fig. 1:**
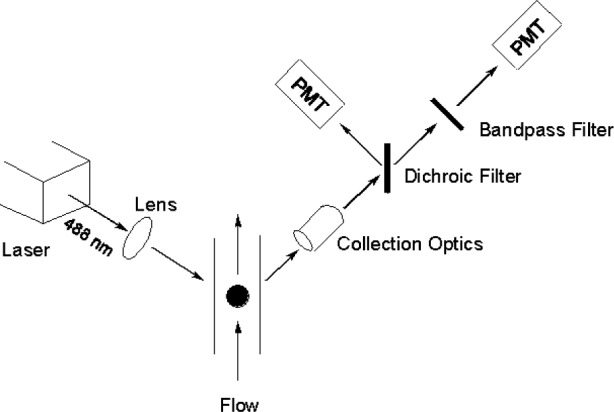
The schematic diagram of the research flow cytometer. An air-cooled Ar ion laser operated at 488 nm is used as the excitation source. The design of the flow system has been described in detail by Shapiro [10].

A flow cytometer is an instrument capable of measuring the fluorescence of fluorescently labeled particles and biological cells one at a time [10]. Because of the design of the flow system in cytometers, fluorescence of the individual cell type, such as lymphocytes, can be measured and quantified in the presence of other cell types. However, such measurement can not be accomplished using typical spectrofluorimeters unless the cells of interest are physically separated from other cell types. In the present study, two flow cytometers were used to obtain MESF values of stained human lymphocytes. One is a FACScan™ flow cytometer from BD Immunocytometry Systems (San Jose, CA), and the other is a custom research cytometer with only two channels, a side scatter channel and a fluorescein fluorescence channel. A schematic of the research cytometer is given in Fig. 1. By comparison, the FACScan flow cytometer is more sophisticated with two scattering channels and three fluorescence channels. The operating procedures for both cytometers are similar except that QC3™ (FITC/PE/PE-Cy5, fluorescein-isothiocyanate/phycoerythrin/phycoerythrin-Cy5) beads (Bangs Laboratories, Inc., Fishers, IN) are run in the FACScan cytometer for the purpose of multi-channel instrumental quality control. The voltage of the photomultiplier tube (PMT) for the fluorescence channel was kept the same for the cell measurements as for MESF microbead measurements in both instruments. However, the PMT voltage for the scattering channel was adjusted for different analytes.

### 2.2 Modeling Methodology

The assignment of MESF values to the beads, which is accomplished using a calibrated spectrofluorimeter, is independent of the spectral properties of the fluorophore in the reference solution compared to when the fluorophore is bound to the bead. This is ensured by integrating over the entire corrected emission spectra of the fluorophore in solution and on the bead. However in the bead case, the measuring instrument, such as a cytometer, may sample over a limited range of emission wavelengths using various bandpass filters. Furthermore the instrument response will usually not be corrected for spectral response. The response of an instrument employing a PMT or charge coupled device (CCD) at a specific wavelength can be modeled as:

$$R(\lambda )=AN\varepsilon_{\text{ex}}\phi Q(\lambda )s(\lambda )T(\lambda ),$$ (1)

where *A* includes illumination, sensing volume, and other wavelength independent parameters associated with the detection system [6]. Since the instrument is a flow cytometer, *N* represents the number of fluorophore on the particle passing the detection region. The parameters, ***ε***_ex_ and *ϕ*, are the extinction coefficient at the excitation wavelength and the fluorescence quantum efficiency, respectively. The function *Q*(λ) describes the relative spectral response of the detector, *s*(λ) is the relative emission function, and *T*(λ) describes the optical filter used in the instrument. In the present study, the responsivity of the detection system [6] has been separated in two parts, the relative spectral response of the detector *Q*(λ) and the optical filter *T*(λ), based on the applications using flow cytometers (Fig. 1). To quantify the response of a biological cell, we find a bead such that the fluorescence intensities of the cell and the bead are equal. The equality of the two signals is modeled by the following equation [6]:

$$\begin{array}{l} \;N_{C}\varepsilon_{\text{ex},C}\phi_{C}\int Q(\lambda )s_{C}(\lambda )T(\lambda )d\lambda \\ =N_{b}\varepsilon_{\text{ex},b}\phi_{b}\int Q(\lambda )s_{b}(\lambda )T(\lambda )d\lambda , \end{array}$$ (2)

where the subscripts C and b stand for cell and calibration bead, respectively. The above equation holds for the situation where the cell and the bead give the same response on the instrument. The equality of the two fluorescence signals is used to assign the same MESF value to the cell as the bead. Equation (2) points out that MESF assignments to biological cells depend on the instrument properties *Q* and *T*. This could lead to problems in assignment of MESF values since different instruments would give different assignments. The instrument factors drop out of Eq. (2) only if the fluorophores on the analyte and bead have the same spectral functions *s*(λ). Moreover, if the extinction coefficient is assumed to be the same for the fluorophore immobilized on both the beads and the cells, Eq. (2) reduces to an equality of fluorescence yields (products of bead or cell number and fluorescence quantum yield).

In the following we present estimates of the error in the assigned MESF values if the conditions that are necessary for rigorous (ideal) application of the MESF concept are not met. Equation (2) will serve as the model of the expected response. We assume that the same instrument settings are used for the cell and bead measurements, and ignore the variation of *Q*(λ) over the narrow range of wavelengths passed by the bandpass filter [*T*(λ) > 0]. We investigate in detail the consequences of the optical filters and mismatch of emission spectra and extinction coefficients.

## 3. Results

To demonstrate the correct use of MESF values and appropriate usage of Fluorescein Solution Standard Reference Material (SRM) 1932 (approximately 60 μM fluorescein dissolved in 0.1 M borate buffer, pH 9.1), a NIST fluorescence standard reference solution, we chose fluorescein as the model fluorophore for the study. It is well known that fluorescence of fluorescein depends highly on its microenvironment [11, 12], thus the logic of the choice is that if one knows how to deal with the complexities of fluorescein, then the same methodologies can be extended to other fluorophores.

**Fig. 2:**
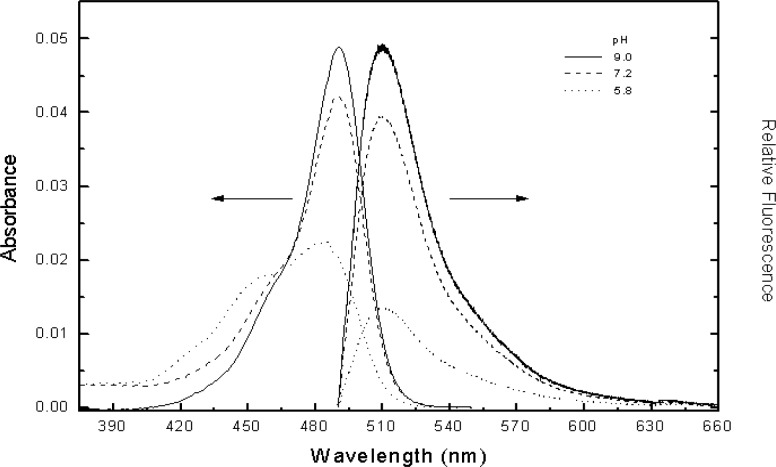
Absorption (left) and emission (right) spectra of fluorescein taken at different pH buffer solutions. The absorbance and fluorescence were measured using a HP-8453 spectrophotometer and the calibrated spectrofluorimeter described in Ref. [6] with an Ar ion laser at 488 nm as the excitation source, respectively.

Figure 2 shows how absorbance and fluorescence of fluorescein change with solution pH. According to Kubista [11] and Sawyer [12], seven protolytic forms of fluorescein exist for solution pH values ranging from 0 to 10. Only two forms exhibit fluorescence with a quantum yield of 0.93 for the fluorescein dianion and 0.37 for the monoanion. As shown in Fig. 2, fluorescence intensity decreases as solution pH value decreases. There is no detectable change in the shape of the emission spectrum with pH. The absorption spectrum shape, however, changes significantly with pH. The changes in absorption and emission spectra with solution pH are consistent with the two reports [11, 12]. While taking fluorescein in borate buffer at pH 9.1 with a quantum yield of 0.93 as the reference, the relative quantum yields for fluorescein in PBS at pH 7.2 and in 2-4-Morpholino-Ethane Sulfonic (MES) buffer at pH 5.8 were determined to be 0.88 and 0.62, respectively. Fluorescein dianion and monoanion species coexist in PBS at pH 7.2 at 91 % and 9 %, respectively, based on the two quantum yields given in the literatures [11, 12].

**Fig. 3:**
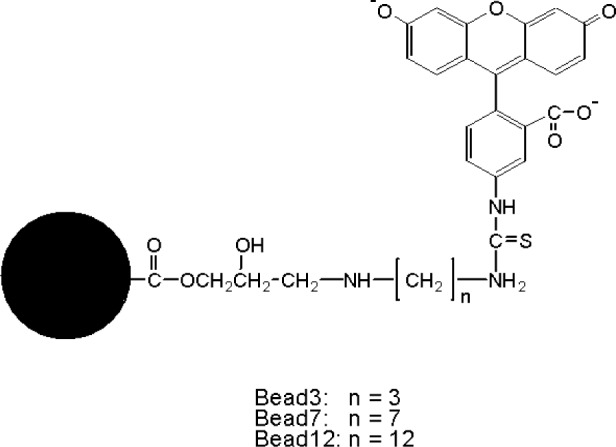
The chemical structures of the fluorescein microbeads with different linker lengths. The abbreviations are also given for three different beads, used throughout the study.

**Fig. 4:**
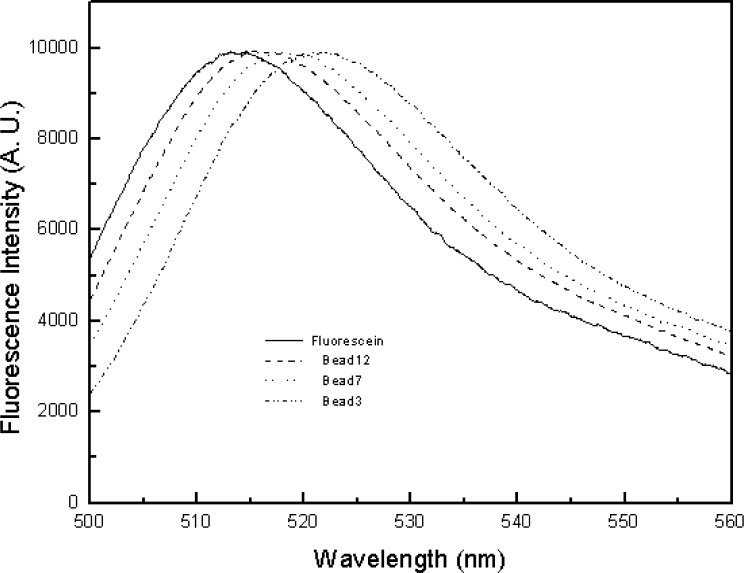
The emission spectra of fluorescein microbeads with different linker lengths in PBS, at pH 7.2, with respect to that of fluorescein in borate buffer solution at pH 9.1. The spectra are normalized to give approximately the same intensities at the maximum. Note that bead3 is the current microbead standard for quantifying fluorescence signal from biological cells stained with fluorescein-labeled monoclonal antibodies. Such a bead standard was produced originally by Flow Cytometry Standards Corporation, San Juan, Puerto Rico, and now by Bangs Laboratories, Inc., Fishers, IN.

In principle, a reference standard for calibrating flow cytometers should have similar size as biological analytes such as fluorescently stained lymphocytes. The fluorophores immobilized on the reference material and biological cells should also experience similar microenvironments. Since polymer microbeads (about 7.2 μm in diameter) with fluorescein covalently attached have been developed and used to calibrate flow cytometers for over a decade, we have adopted the microbead approach with a systematical evaluation of the methodology. The investigations are aimed to build a foundation for fluorescence quantitation and to demonstrate the merits of a particular fluorescence reference material. The chemical structures of fluorescein-labeled microbeads used in the study are shown in Fig. 3 with different linker lengths^2^. When fluorescein is immobilized on the surface of the microbeads, its emission maximum shifts towards the red with respect to that of fluorescein in solution (Fig. 4). The shift is sensitive to the length of the linker. The shorter the linker, the greater this bathochromic shift. A modified Lippert equation [13] was used in a separate study to correlate the spectral shifts in terms of the dielectric properties of the solvent and polymer bead materials (the result is not published yet).

**Fig. 5:**
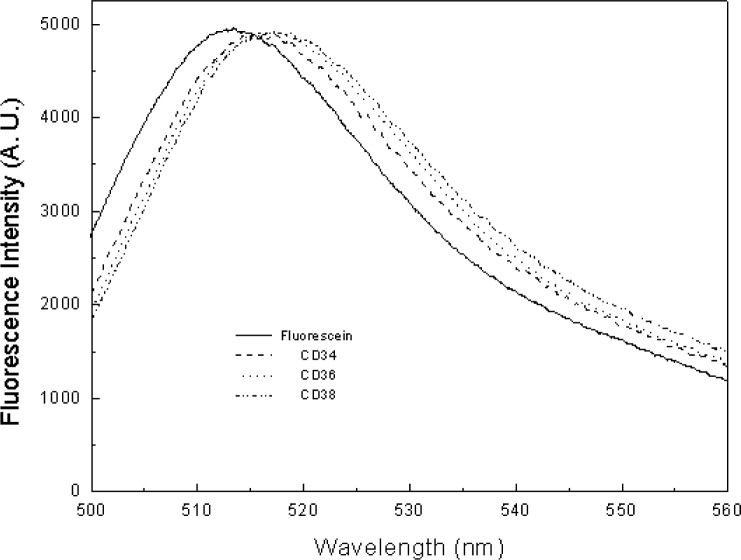
The normalized emission spectra of three fluorescein-labeled monoclonal antibodies from three different manufacturers in PBS, pH 7.2, relative to that of fluorescein in borate buffer solution at pH 9.1. The three antibodies are CD34 (BD Bioscience, San Jose, CA), CD36 (AMAC INC., Westbrook, Maine), and CD38 (Caltag, Burlingame, CA).

**Fig. 6:**
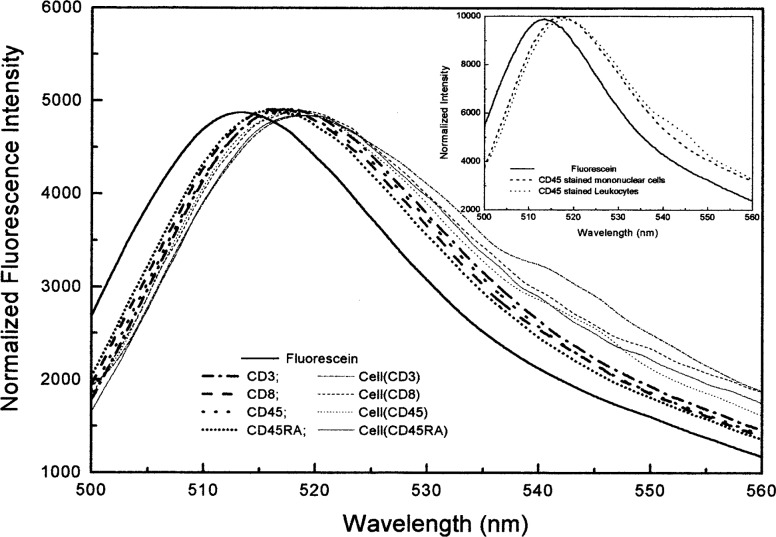
The normalized emission spectra of fluorescein-labeled monoclonal antibodies (thick plots) and leukocytes stained with these antibodies (thin plots) in PBS, pH 7.2, relative to fluorescein in borate buffer, pH 9.1: CD3; CD8; CD45; CD45RA. The inset shows the normalized spectra of CD45 stained leukocytes and mononuclear cells compared to that of fluorescein in solution.

Figure 5 shows the emission spectra of three fluorescein-labeled monoclonal antibodies from different vendors compared with that of fluorescein in borate buffer. About 30 fluorescein-labeled monoclonal antibodies have been measured, including the same antibody produced by different companies and by the same company but in different lots. There is little spectral shift among these antibodies, but the spectra are shifted to the red when compared to fluorescein in solution. When leukocytes were stained with several fluorescein-labeled antibodies, CD3, CD8, CD45, and CD45RA, the spectra of the stained cells shift further to the red with respect to those of the antibodies (Fig. 6). The reason for choosing these four antibodies is that the surface receptors for the four antibodies on leukocytes are relatively abundant compared to those for other antibodies in the study. Therefore, it’s relatively easy to measure their emission spectra using the calibrated spectrofluorimeter with low laser power. The spectra of stained leukocytes show large deviation from fluorescein, especially CD3-stained cells. Since the measurements were carried out with low power from an Ar ion laser (≤ 0.5 mW) and a flow cell to minimize photodegradation effects, any other light source including room light would contribute to the spectral background which might not be subtracted fully due to the heterogeneity of leukocyte suspensions. In the inset of Fig. 6, the emission spectrum of CD45-stained mononuclear cells is shown to match well with that of the same antibody-stained leukocytes. Unlike leukocytes, mononuclear cells include lymphocytes and monocytes, but exclude granulocytes. The measurement further demonstrates that the obtained spectra can best represent that of pure lymphocytes. Historically, lymphocytes have been the focal interest in cytometric measurements.

**Fig. 7:**
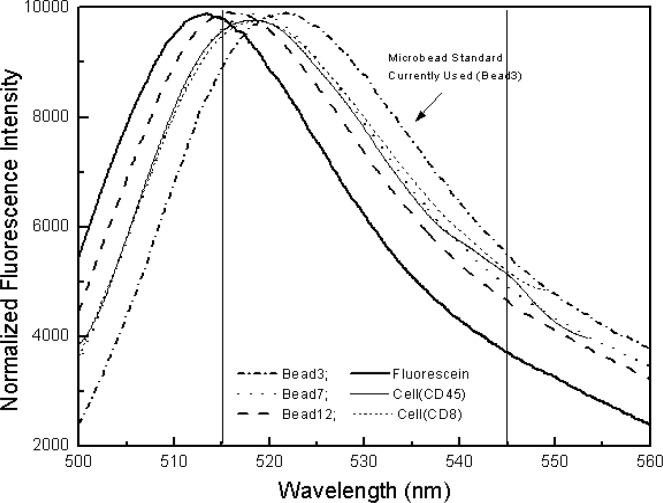
The normalized emission spectra of the microbeads with different linker lengths and leukocytes stained with either CD45 or CD8 monoclonal antibody, with respect to that of fluorescein in solution: Fluorescein; Bead3; Bead7; Bead12; cell(CD45); cell(CD8). The two vertical lines define the emission collection window by the bandpass filter used in flow cytometers.

Figure 7 displays the spectral comparison between fluorescein in solution, microbeads with different linker lengths, and stained leukocytes. The figure includes the emission spectrum of bead3, a commercially available and widely used calibration standard for flow cytometers. For the purpose of visualization, leukocytes stained with CD45 and CD8 are also shown, and their spectra match most closely with that of bead7. In the following, we will use the commercial calibration bead, bead3, to perform the assignments of MESF values to the lymphocytes stained with fluorescein-labeled CD45 monoclonal antibodies using two different flow cytometers as described earlier. Mathematical modeling is used to assess the systematic error associated with the spectrum mismatch between bead3 and lymphocytes and the optical components used in the flow cytometers.

**Fig. 8:**
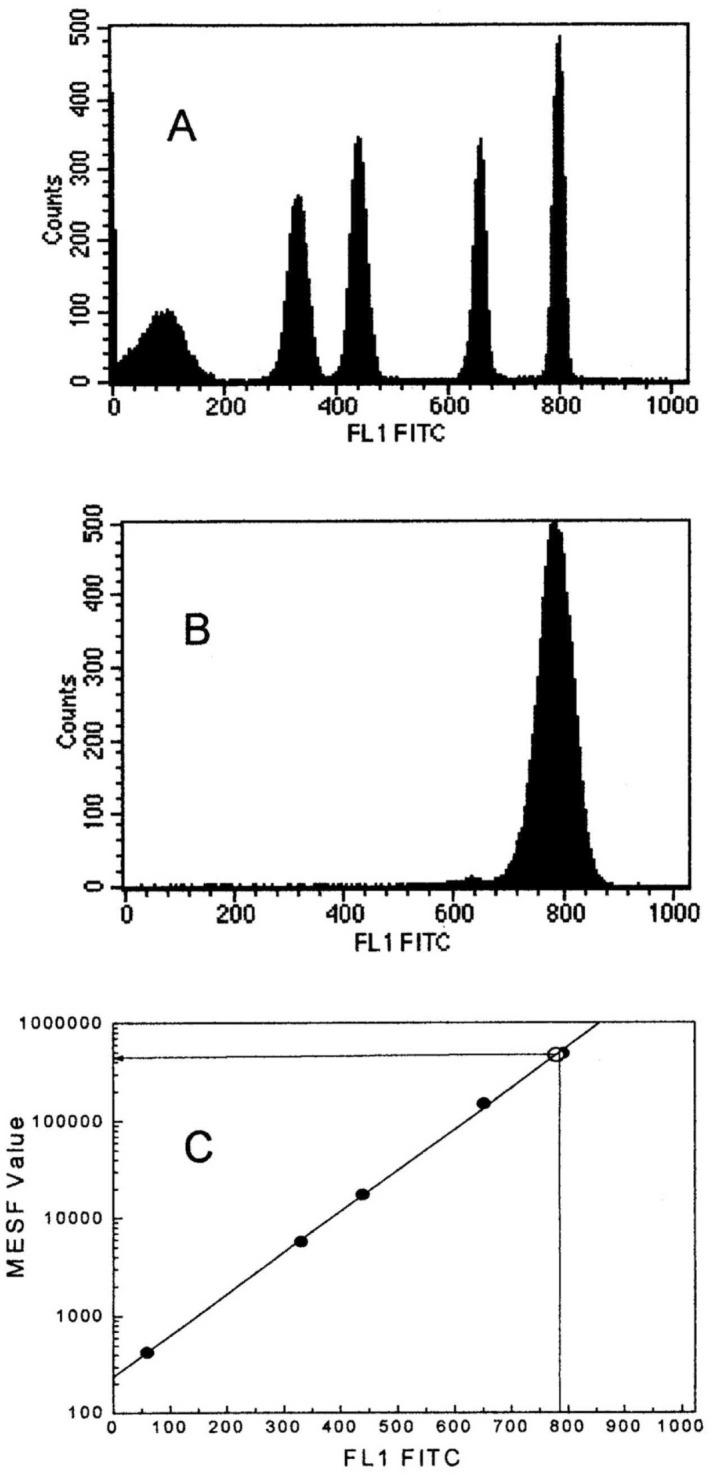
The method of assigning a MESF value to lymphocytes stained with fluorescein-labeled CD45 monoclonal antibodies. (A) Histogram of four populations of the fluorescein-labeled microbeads (sharp peaks) and one blank bead (broad peak) obtained by the FACScan flow cytometer. FL1 FITC refers to the fluorescein fluorescence channel. (B) Histogram of CD45 stained lymphocytes. (C) A calibration curve of MESF value vs fluorescence intensity in terms of the fluorescence channel number obtained through the linear fitting of the mean channel numbers for the five-microbead populations (solid circles). Having a known mean channel number for the stained lymphocytes (open circle), the corresponding MESF value is determined.

Figure 8 gives an example of how to derive a calibration curve and to make a MESF assignment to CD45-stained lymphocytes. Figure 8A displays the intensities of four MESF beads and one blank bead in term of counts in the green fluorescence channel. The acquisition software, CELLQuest™ from BD Immunocytometry Systems (San Jose, CA) gives the mean of the individual population assuming a log normal distribution. In Fig. 8C we see the mean values determined by the measurement serving as the x axis, and the known MESF values of the four fluorescein-labeled beads provided by the manufacturer serving as the y axis, to obtain the calibration curve using a program named QuickCal^®^ (Flow Cytometry Standards Corporation, San Juan, PR). Figure 8B shows the histogram of CD45-stained lymphocytes. The same analysis provides the mean channel number of the stained cells. Thus, its position on the calibration curve is located (Fig. 8C). The horizontal line points to the MESF value of the stained lymphocytes. We performed an experiment to determine the MESF values of the same lymphocytes stained with fluorescein-labeled CD45 antibodies using the two cytometers specified in the experimental section. The MESF values were 500646 with a Coefficient of Variation (CV) [10] of 1.76 % for the research cytometer and 477079 (CV, 0.39 %) for the FACScan flow cytometer^3^. Both CVs were obtained from four consecutive measurements of the same sample. The two MESF values differ by 4.9 %.

In the following, we present estimates of the error in the assigned MESF values based on Eq. (2). The research cytometer (Fig. 1) serves as the model cytometer because it’s easy to model. We ignore the variation of *Q*(λ) given that a Hamamatsu R3896 PMT is employed as the fluorescence detector, and this PMT shows a negligible variation of the spectral response over the narrow range of wavelengths (515 nm ≲ λ ≲ 545 nm) passed by the bandpass filter, centered at 530 nm with bandwidth at half height of 30 nm. The filter transmits approximately 80 % of light with a ± 5 % variation in the range of wavelengths specified. Thus, we also ignore the deviation (± 5 %) caused by the bandpass filter. The consequences of the dichroic filter, mismatch of emission spectra reflected in *s*(λ), and disparity of the extinction coefficients are examined.

**Fig. 9:**
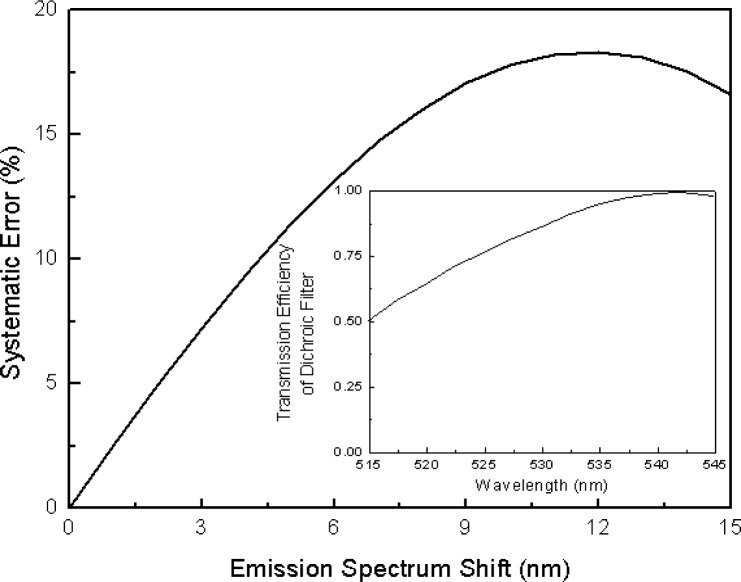
Graph of the systematic error as a function of the emission spectrum shift relative to that of mononuclear cells using the simple research cytometer as the model instrument. The inset shows the change in the transmission efficiency of the dichroic filter used in the model cytometer in the wavelength range from 515 nm to 545 nm.

The inset of Fig. 9 exhibits a change in the transmission efficiency of the dichroic filter over the wavelength range from 515 nm to 545 nm. We have chosen the emission spectrum of CD45-stained mononuclear cells shown in the inset of Fig. 6 as the model spectrum because it best represents that of the lymphocytes in the cytometric measurements. Using Mathcad 2001 software from MathSoft Inc., Cambridge, MA, the “cspline” function was used to mimic the spectrum of the stained cells. By shifting the model spectrum to the red where the emission spectrum of the currently used calibration standard, bead3, stands, the possible error due to the spectral shift combined with variation in transmission of the dichroic filter in the observation window was modeled (Fig. 9). The error for a given shift results from the integration term shown in Eq. (2) and depends on the specific instrument used.

**Fig. 10:**
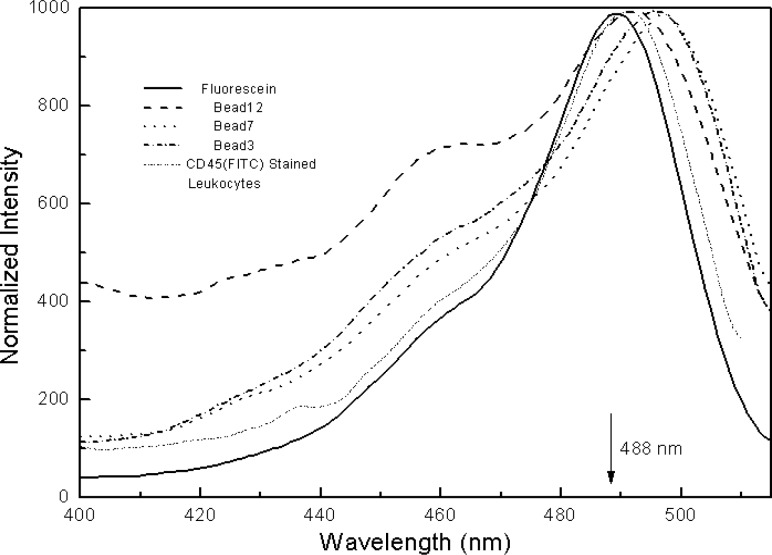
The normalized excitation spectra of the microbeads with different linker lengths and leukocytes stained with fluorescein-labeled CD45 monoclonal antibodies in PBS, pH 7.2, with respect to that of fluorescein in borate buffer, pH 9.1. The background from the corresponding blank beads or unstained leukocytes has been subtracted. The emission was collected at 530 nm with the excitation bandwidth of 2 nm and the emission bandwidth of 5 nm. The excitation and emission polarizers were set at 0° and 54.7°, respectively.

It’s well known that measurement of the extinction coefficient in a highly scattering environment is extremely challenging [14]. In our case, for example, the absorption spectrum of bead12 could not be obtained with satisfaction by simple subtraction of the absorbance from the blank bead12 to which no fluorescein was attached. The attachment of fluorescein to the outer surface of the microbead results in significant changes in the transmitting and scattering properties of the bead. Given that the absorption of fluorescein attached to the bead should be proportional to the fluorescence signal obtained at the same excitation wavelength when the emission wavelength is held constant, we measured the excitation spectra of the beads and CD45-stained cells with respect to fluorescein in solution. The excitation spectra shown in Fig. 10 are normalized. To our surprise, the excitation (Fig. 10) and emission (Fig. 4) spectra of the microbeads lack a mirror-image relationship. The maximum of the excitation spectrum of bead7 slightly shifts to the red compared to that of bead3. The experiment was repeated, and the same results were obtained. Figure 10 also shows the excitation spectrum of CD45-stained leukocytes. The spectrum mimics that of fluorescein in solution except that the spectral shape expands toward the red. The spectral shapes of various beads are also wider than that of the stained cells although the same PBS is used as the medium for the measurements. If we assume that these spectra represent the profiles of relative extinction coefficients of beads and cells, the extinction coefficient of bead3 at 488 nm is 7.5 % lower than that of the stained leukocytes. In other words, the MESF assignment of the cells based on bead3 as the calibration standard will cause as much as a 7.5 % error in the assigned value.

## 4. Discussion

As shown in Fig. 1, fluorescein fluorescence is very sensitive to the solution pH. To serve as a calibration standard for flow cytometers, the microbeads labeled with fluorescein need to be suspended in the same buffer solution as biological cells to maintain the similar microenvironments for the fluorophore. Since PBS, pH 7.2, is commonly used for biological cells, it should be used to make bead and cell suspensions. On the other hand, Fluorescein Solution SRM 1932 is made of fluorescein dissolved in borate buffer, pH 9.1. Since two fluorescein species exist in PBS with different quantum yields and only the fluorescein dianion is present in borate buffer solution, MESF values can not be assigned to microbeads suspended in PBS using the calibration curve made by a series of dilutions of Fluorescein Solution SRM in borate buffer. In the following we present the measurement model which provides a framework for clarifying pH dependence of the MESF assignments. The superscript “s” refers to properties in solution and the superscript “b” refers to properties of fluorescein on the bead. Thus, *f*^s^ and *f*^b^ will give the fraction of fluorescein in the dianion form in solution and on the bead, respectively. The equality of fluorescence radiance from a solution and suspension, both at some specified pH, gives the following equality:

$$\begin{array}{l} \;\varepsilon_{d}^{b}\phi_{d}^{b}f^{b}N^{b}+\varepsilon_{m}^{b}\phi_{m}^{b}(1-f^{b})N^{b}\\ =\varepsilon_{d}^{s}\phi_{d}^{s}f^{s}N^{s}+\varepsilon_{m}^{s}\phi_{m}^{s}(1-f^{s})N^{s} \end{array}$$ (3)

where the subscripts “d” and “m” refer to dianion and monoanion forms of fluorescein. *N*^b^ and *N*^s^ are the number concentrations of beads in suspension and fluorescein molecules in solution, respectively. The MESF values are assigned using a specified series of operations. The fluorescein concentration in solution is varied until the normalized fluorescence radiance is the same as that of the bead suspension. Then the ratio of solution concentration to bead concentration yields the MESF value for the bead. Assuming the extinction coefficients of the fluorescein dianion in solution and on beads are equal, Eq. (3) can be used to obtain the MESF value for the bead.

$$\begin{array}{c} MESF^{\text{Buffer}}={\left(\frac{N^{s}}{N^{b}}\right)}^{\text{Buffer}}\\ ={\left\{\frac{\phi_{d}^{b}\left[f^{b}+\frac{\varepsilon_{m}^{b}\phi_{m}^{b}}{\varepsilon_{d}^{b}\phi_{d}^{b}}(1-f^{b})\right]}{\phi_{d}^{s}\left[f^{s}+\frac{\varepsilon_{m}^{s}\phi_{m}^{s}}{\varepsilon_{d}^{s}\phi_{d}^{s}}(1-f^{s})\right]}\right\}}_{\text{MODEL}}^{\text{Buffer}} \end{array}$$ (4)

where the ratio of concentrations is obtained from measurements and the quantity in brackets is an estimate of the ratio of concentrations of fluorescein in the two environments with two ionic forms. For the case of borate buffer, pH 9.1, *f*^s^ = 1 and *f*^b^ ≅ 1. Eq. (4) reduces to

$$MESF^{\text{Borate}}={\frac{N_{s}}{N_{b}}}^{\text{Borate}}={\left(\frac{\phi_{d}^{b}}{\phi_{d}^{s}}\right)}_{\text{MODEL}}^{\text{Borate}}$$ (5)

which corresponds to the idealized case discussed in the previous paper [6]. The ratio of concentrations is the ratio of quantum yields. Since the quantum yield is higher in solution we see that the model predicts that there will be fewer soluble fluorophores needed to give the same fluorescence yield as the bead. If the MESF assignment is performed in PBS, pH 7.2, Eq. (4) gives the expected change in MESF values

$$MESF^{\text{PBS}}=MESF^{\text{Borate}}{\left[\frac{f^{b}+\frac{\varepsilon_{m}^{b}\phi_{m}^{b}}{\varepsilon_{d}^{b}\phi_{d}^{b}}(1-f^{b})}{f^{s}+\frac{\varepsilon_{m}^{s}\phi_{m}^{s}}{\varepsilon_{d}^{s}\phi_{d}^{s}}(1-f^{s})}\right]}^{\text{PBS}}$$ (6)

Here we reasonably assume that

$${\left(\phi_{d}^{b}/\phi_{d}^{s}\right)}^{\text{Borate}}={\left(\phi_{d}^{b}/\phi_{d}^{s}\right)}^{\text{PBS}}.$$ (7)

**Fig. 11:**
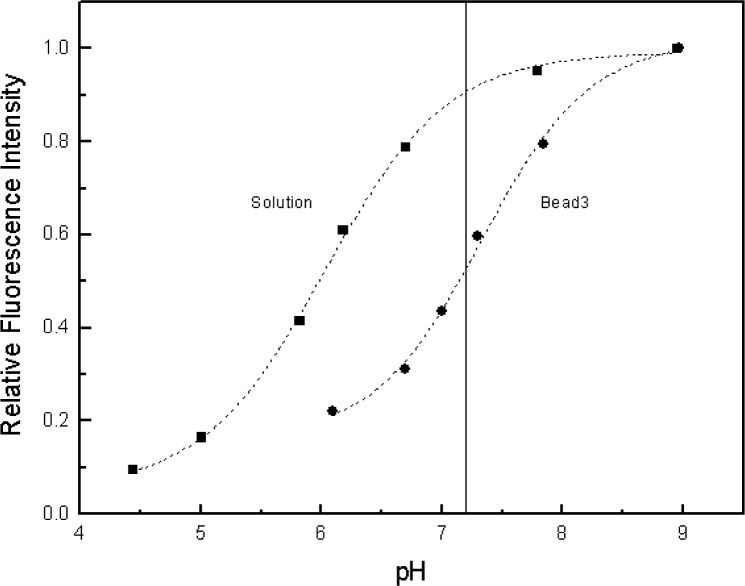
The fluorescence intensity relative to the intensity at pH ≈ 9.0 as a function of the pH of the medium for fluorescein (solid square) and bead3 labeled with fluorescein (solid circle). Dashed lines are the sigmoidal fitting curves for the two sets of data.

The internal conversion processes predominantly affect quantum yield of fluorescein through changes in molecular symmetry. The quantum efficiencies of a single fluorescein species, fluorescein dianion, in solution and on bead would not depend on the pH of the medium. In Eq. (6) the values, *f*^s^ and *f*^b^, will be different in PBS if the protonation equilibrium is different on the bead surface from that in solution. Figure 11 shows the pH titration curves for both fluorescein in solution and bead3 conjugated with fluorescein. The protonation equilibrium for the beads shifts to higher pH by about one pH unit compared to that for fluorescein in solution. This indicates that at pH 7.2 *f*^s^ > *f*^b^, meaning the dianion form is more dominant in solution relative to the bead form. Such differences are expected due to surface charge, steric effects, and transport differences. We have obtained calibration curves (fluorescence intensity vs concentration) for Fluorescein Solution SRM diluted in borate buffer and in PBS. These two curves are used to assign MESF values to bead3 suspended in borate buffer and in PBS, respectively. The ratio of the MESF values assigned for the same bead3 in PBS and in borate buffer [Eq. (6)] is 0.76. The same experiment was repeated for bead3 labeled with a different amount of fluorescein, and the ratio was found to be 0.72. It appears experimentally that the quantity in the bracket in Eq. (6) is close to a constant for bead3 labeled with different amounts of fluorescein. This result is consistent with the fact that the quantity in the bracket in Eq. (6) is determined by the molecular properties of fluorescein and bead3 at pH 7.2, and should be a constant. Since PBS, pH 7.2, is the preferred medium for the biological cells, it is a good practice to determine MESF values of biological cells in PBS, pH 7.2, using assigned MESF microbeads (bead3) in PBS, pH 7.2, using flow cytometers. This ensures that fluorescein molecules conjugated to cells and beads experience similar microenvironments. Equation (6) points out that any change in the pH value of the medium will give rise to deviation in the assigned MESF values using flow cytometers.

In the previous paper, emission spectrum matching between beads and biological cells was emphasized so that the MESF values assigned to cells by flow cytometers will be instrument independent [6]. We have found in the present study that the emission spectrum of bead7 matches those of stained cells the best (Fig. 6). Relative to bead7 and stained cell spectra, the spectrum of bead3, which is widely used as the cytometric calibration standard for fluorescein based assays in clinical and research laboratories, shifts to the red by approximately 5 nm. The spectrum shift of bead3 with respect to that of biological cells causes the MESF value assigned to cells to be lower by 11.5 % using the research flow cytometer as the model instrument (Fig. 9). The error is attributed to both the spectral mismatch between cells and bead3 and transmission efficiency variation of the dichroic filter in the observation window. It’s worthy of mention that commercial instruments, FACScan cytometer from BD Immunocytometry Systems as an example, have more complex and sophisticated optical components with various efficiencies within the observation window (515 nm/545 nm). Thus, the systematic error may be larger than shown here. Importantly the systematic error due to the spectrum shift can be minimized with the use of bead7 as the calibration standard.

Using the existing microbead calibration standard, bead3, the MESF values of CD45-stained lymphocytes were determined and compared. The MESF value determined using the research cytometer is 4.9 % higher than that obtained using the FACScan cytometer as described in the results section. To verify the source of fluctuation in the assigned MESF values, this experiment was repeated. The values obtained were 407519 (CV, 1.83 %) for the research cytometer and 465156 (CV, 0.44 %) for the FACScan cytometer. The MESF value determined by the research cytometer is 12.4 % lower than that of the commercial cytometer. These two experiments clearly show the existence of procedure error given that the systematic errors associated with two flow cytometers are similar in the two experiments. Vogt et al. have reported the error in the interlaboratory study of cellular fluorescence intensity measurements made on 43 different flow cytometers in 34 laboratories [1]. In their studies, the same fluorescein-labeled microbead calibrator and biological samples were used in all 34 laboratories, and the CV in the assigned MESF was found to be 24 %. This error is highly related to measurement procedures because the systematic error should be similar between different laboratories with the use of the same microbead calibrator and biological samples. The flow cytometers from different manufacturers follow different quality control procedures recommended by the manufacturers for performing the measurements. This step attributes to the procedure error of the measurements. Hence, standardizing the measurement procedures would be an important step subsequent to reducing the systematic error to reach the goal of quantitative flow cytometry.

When MESF values obtained by two different cytometers are compared, we assume that the extinction coefficients of fluorescein on the surfaces of beads and lymphocytes are equal. In fact, the beads and lymphocytes may not absorb equally at 488 nm as seen from their peak-normalized excitation spectra in Fig. 10. The excitation spectrum is proportional to the absorption spectrum assuming that the relative emission function, *s*(λ), does not change over the wavelength range. It’s possible to obtain relative extinction coefficients of fluorescein on beads and cells through fluorescence measurements. However, one has to be extremely careful about issues like background subtraction and measurement methodology. As shown in Fig. 10, the signals of bead12 at lower wavelengths (λ ≤ 470 nm) are much higher than those of bead3 and bead7 although the background from blank bead12 has been subtracted. The large signal is likely due to interactions between fluorescein molecules and between fluorescein and the polymer surface. The long linker may allow fluorescein molecules to interact and form complexes [15]. For turbid bead and biological cell suspensions, front-face fluorescence measurement may be implemented to decrease inner filtering effects [13]. On the other hand, recent reports point out that measurements of resonance light scattering combined with absorbance leads to the determination of extinction coefficients of the fluorophores that form particles through self-assembly over the wavelength range measured in the scattering environment [16, 17]. The absorption spectrum of a turbid solution, as measured by a conventional spectrophotometer, is the sum of two extinction components due to absorption and scattering, respectively. Light scattering measurements can be performed on a spectrofluorimeter in the synchronous scanning mode in which the emission and excitation monochromators are preset to identical wavelengths. This allows the scattering profile of the solution to be obtained and used to correct the scattering intensity from the absorption spectrum. In our case, fluorophores occupy only a fraction of the surface area; therefore, care has to be taken to correct for optical field distortion near the particle surface.

In summary, the emission spectrum matching between calibration microbeads and biological cells is extremely important. It ensures that the instrument dependence of the measurements is minimized. Furthermore, accurate measurements of the extinction coefficients of beads and cells at the excitation wavelength of flow cytometers, usually 488 nm, are equally valuable. Until these two prerequisites are met, fluorescence quantitation in cytometric measurements can not be accomplished.

## 5. Conclusions

The present investigation is an extension of the conceptual framework for assigning a MESF value for a suspension in terms of a reference solution. The goal of the study is to address crucial steps to make MESF assignments of biological cells as accurate as possible. As described in the earlier paper [6], the MESF assignments can be made to suspensions of beads to which fluorophores are attached using a reference solution and a calibrated spectrofluorimeter. Since the instrument allows the integration of the whole emission spectrum, the types of fluorophores used in the reference solution and the suspension can be different as long as the normalization of their extinction coefficients at the excitation wavelength is applied. In measurements using flow cytometers, however, only a limited range of emission wavelengths is sampled because of the use of a bandpass filter. In this case, emission spectrum matching between a reference and an analyte becomes extremely important. The present study points out an 11.5 % error caused by a mismatch of the two spectra using the simple research cytometer as the model instrument and fluorescein as the model fluorophore. Since fluorescein fluorescence depends on solution pH, MESF assignments must be made under the same pH condition. In regard to spectrum matching, the work also shows that the emission spectrum of bead7 matches those of biological cells better than bead3, the calibration standard currently used for flow cytometers. To better ensure that measurements are instrument independent, use of bead7 as the reference standard is advised. Furthermore, we have emphasized the importance of accurate extinction coefficient measurements. Based on the excitation measurements of the apparent extinction coefficients of bead3 and cells, the MESF values assigned to the cells could be lower by as much as 7.5 %. Combining the two errors caused by spectral mismatch and the discrepancy in the extinction coefficients, the systematic error in assigned MESF values is approximately 18.1 % using bead3 as the calibration standard. The statistical error associated with the assignments is less than 1 %. Although it’s logical to assume equal extinction coefficients with use of the same fluorophore in reference solution and analytes, accurate measurements are crucial for reliable quantitative flow cytometry. While fluorescein is used throughout the investigation because of the availability of the Fluorescein Solution SRM, the same principle of MESF assignment can be applied to other fluorophores. The MESF unit as a practical methodology for quantifing fluorescence signal can also be applied to other fluorescence-based assays, such as DNA microarrays.

## Footnotes

1 Certain commercial equipment, instruments, or materials are identified in this paper to foster understanding. Such identification does not imply recommendation or endorsement by the National Institute of Standards and Technology, nor does it imply that the materials or equipment identified are necessarily the best available for the purpose.

2 Fluorescein-labeled microbeads are obtained through the labeling reaction between fluorescein-6-isothiocyanate (FITC) and amine-functionalized microbeads. So are the fluorescein-labeled monoclonal antibodies used in the study.

3 These MESF values were determined through gated analysis [10] and without compensation [10].
